# Supplementary material for: Dental Implants in People with Osteogenesis Imperfecta: A Systematic Review
Source: Int J Environ Res Public Health. 2022 Jan 29;19(3):1563. doi: 10.3390/ijerph19031563 (PMC8835393; doi:10.3390/ijerph19031563)
Supplement: Supplementary file 1 [file ijerph-19-01563-s001.zip › Table S1. QualityAssessment.pdf]

Quality – Assessment (Murad MH, Sultan S, Haffar S, *et al* Methodological quality and synthesis of case series and case reports *BMJ Evidence-Based Medicine* 2018;**23:**60-63.)

Domains	Leading explanatory questions
Selection	1. Does the patient(s) represent(s) the whole experience of the investigator (center) or is the selection method unclear to the extent that other patients with similar presentation may not have been reported?
Ascertainment	2. Was the exposure adequately ascertained?<br>3. Was the outcome adequately ascertained?
Causality	(4. Were other alternative causes that may explain the observation ruled out?<br>5. Was there a challenge/rechallenge phenomenon?<br>6. Was there a dose–response effect?)<br>7. Was follow-up long enough for outcomes to occur?
Reporting	8. Is the case(s) described with sufficient details to allow other investigators to replicate the research or to allow practitioners make inferences related to their own practice?

Questions 4, 5 and 6 are mostly relevant to cases of adverse drug events.

We suggest using this tool in systematic reviews of case reports/series. One option to summarise the results of this tool is to sum the scores of the eight binary responses into an aggregate score. A better option is not to use an aggregate score because numeric representation of methodological quality may not be appropriate when one or two questions are deemed most critical to the validity of a report (compared with other questions). Therefore, we suggest making an overall judgement about methodological quality based on the questions deemed most critical in the specific clinical scenario.

**Study**	Binger T, Rücker M, Spitzer WJ. Dentofacial rehabilitation by osteodistraction, augmentation and implantation despite osteogenesis imperfecta. Int J Oral Maxillofac Surg. June 2006;35(6):559–62.	Yes<br>No<br>Partially	Text reference	Rationale
**Selection**				
Does the patient(s) represent(s) the whole experience of the investigator (center) or is the selection method unclear to the extent that other patients with similar presentation may not have been reported?		Yes	A 32-year-old woman was referred by her general dental practitioner for pre-prosthetic<br>surgery.	Single case report with no indication that other patients with similar presentation may not have been reported.
**Ascertainment**				
Was the exposure adequately ascertained?		Yes	…five dental implants (ITI dental standard implants, Straumann GmbH, Freiburg, Germany) were inserted…	Implant position is indicated on the x-ray and information regarding the type of implants was provided, therefor the exposure was adequately ascertained.
Was the outcome adequately ascertained?		Yes	Functional and aesthetic rehabilitation with an implant-supported overdenture prosthesis…<br>No loss of bone after 4 years…	Information regarding the bone loss after 4 years was described. Also, information regarding the type of prosthesis on the abutments.
**Causality**				
Were other alternative causes that may explain the observation ruled out?		No	-	No bone loss or loss of implants. Therefore, this domain does not seem to be important for this study.
Was follow-up long enough for outcomes to occur?		Yes	X-ray demonstrating no loss of bone after 4 years.	4 years of follow-up and no bone loss indicate stable situation.
**Reporting**				
Is the case(s) described with sufficient details to allow other investigators to replicate the research or to allow practitioners make inferences related to their own practice?		Yes	Multiple (also see the references above).	Detailed information on surgery procedure and materials used was provided.
**Overall rating**	Good quality of reporting with enough details for treating similar patients alike. Detailed information regarding the type of implants, the surgical procedure and the prosthodontic rehabilitation were stated. A follow-up of 4 years with no loss of bone indicates a stable situation, although longer follow-ups are necessary.			

Study	Caicedo-Rubio M, Ferrés-Amat E, Ferrés-Padró E. Implant-supported fixed prostheses in a Patient with Osteogenesis Imperfecta: A 4-year follow-up. J Clin Exp Dent. December 2017;9(12):e1482–6.	Yes<br>No<br>Partially	Text reference	Rationale
**Selection**				
Does the patient(s) represent(s) the whole experience of the investigator (center) or is the selection method unclear to the extent that other patients with similar presentation may not have been reported?		Yes	The case involves a 61-year-old, male patient, who arrived in the dental clinic because he was not able to chew properly due to his removable prosthesis.	Single case report with no indication that other patients with similar presentation may not have been reported.
**Ascertainment**				
Was the exposure adequately ascertained?		Yes	Implant supported prosthesis on implants […] of MIS Implants Technologies LTD, Shlomi, Israel, two implants area 36-35 […], one in 46 […], MIS Multiunit abutments in third quadrant, MIS transepithelial abutment.	Detailed information on position and type of implants and detailed information on surgical procedure.
Was the outcome adequately ascertained?		Yes	…peri-implant tissues are stable, with a loss of 1.25mm of crestal bone on the area of implant 36, the same level that was measured in 2014.	Stable peri-implant tissues with slight bone loss after 2 years which stayed constant for all 4 years of follow-up.
**Causality**				
Were other alternative causes that may explain the observation ruled out?		Yes	…if he is exposed to OMIB associated risk factors, smoking, bad oral hygiene, periodontal disease.	Alternative causes were stated with the remark that they form possible risk factors.
Was follow-up long enough for outcomes to occur?		Yes	In later follow-ups until July 2016…	4 years and 1,25mm bone loss which stayed constant since 2014 indicates a stable situation.
**Reporting**				
Is the case(s) described with sufficient details to allow other investigators to replicate the research or to allow practitioners make inferences related to their own practice?		Yes	Multiple (also see the references above).	Detailed information on surgery procedure and materials used was provided.
**Overall rating**	Good quality of reporting with detailed information regarding types, positioning, and procedure of implantation. Detailed information regarding the patient's history and supportive information regarding risk factors.			

Study	Friberg B. Brånemark system implants and rare disorders: a report of six cases. Int J Periodontics Restorative Dent. April 2013;33(2):139–48.	Yes<br>No<br>Partially	Text reference	Rationale
**Selection**				
Does the patient(s) represent(s) the whole experience of the investigator (center) or is the selection method unclear to the extent that other patients with similar presentation may not have been reported?		Yes	A 51-year-old woman was referred to the Brånemark Clinic in 2006.	Single case report with no indication that other patients with similar presentation may not have been reported.
**Ascertainment**				
Was the exposure adequately ascertained?		Partially	Six regular-platform Ti-Unite Brånemark System implants were inserted in March 2007.	Information regarding implant position can only be seen on radiographs.
Was the outcome adequately ascertained?		Partially	Despite maintaining perfect oral hygiene during 4 years of follow-up, the marginal bone levels have not been stable.	Detailed information regarding the loss of bone for individual implants is missing and is only described as “not been stable”.
**Causality**				
Were other alternative causes that may explain the observation ruled out?		Yes	…exhibited periodontal disease around the remaining dentition in the maxilla.<br>… perfect oral hygiene during 4 years of follow-up…	Because of perfect oral hygiene, bone loss regarding periodontal disease can be ruled out.
Was follow-up long enough for outcomes to occur?		Partially	…4 years of follow-up…	4 years of follow-up and a situation described as “not stable” may not offer enough information regarding implant survival.
**Reporting**				
Is the case(s) described with sufficient details to allow other investigators to replicate the research or to allow practitioners make inferences related to their own practice?		Partially	Multiple (also see the references above).	More detailed information regarding surgical procedure and bone-loss at time of follow-up are required.
**Overall rating**	Medium quality of reporting. Information regarding implantation site, type of abutment and radiological bone loss is missing. Follow-up may not be long enough to rate implant survival, because an unstable situation is described after 4 years. Risk factors (periodontal disease) were assessed and do not seem to have caused the bone loss, because perfect oral hygiene was described.			

**Study**	Hanisch, M.; Maus, M.; Kleinheinz, J. Implant-Prosthetic Restoration of a Patient with Osteogenesis Imperfecta: A Case Report. International Journal of Environmental Research and Public Health 2021, 18 (8).	Yes<br>No<br>Partially	Text reference	Rationale
**Selection**				
Does the patient(s) represent(s) the whole experience of the investigator (center) or is the selection method unclear to the extent that other patients with similar presentation may not have been reported?		Yes	A 64-year-old otherwise healthy female patient presented for the first time in May 2019 for a specialty consultation concerning rare diseases with oral involvement.	Single case report with no indication that other patients with similar presentation may not have been reported.
**Ascertainment**				
Was the exposure adequately ascertained?		Yes	...two narrow areas were identified in regions 32 and 44, in which the insertion of two tissue-level implants […] was possible without the use of augmentation measures.	Detailed information on position and type of implants and detailed information on surgery procedure.
Was the outcome adequately ascertained?		Yes	...exhibited no signs of […] peri-implant infection.<br>...tested negative for bleeding on probing at both implants…	Stable peri-implant tissue with no signs of bleeding. Probing depths at time of follow-up were stated.
**Causality**				
Were other alternative causes that may explain the observation ruled out?		Yes	…patient´s history of drug therapy with alendronic acid and donosumab.	History of drug therapy was assessed and with no signs of bisphosphonates altering the peri-implant tissue so far.
Was follow-up long enough for outcomes to occur?		Partially	…regular follow-ups for one year…	No sign of significant bone loss after one year, longer follow up time necessary to evaluate implant survival further.
**Reporting**				
Is the case(s) described with sufficient details to allow other investigators to replicate the research or to allow practitioners make inferences related to their own practice?		Yes	Multiple (also see the references above).	Detailed information on surgery procedure and materials used was provided.
**Overall rating**	Good quality of reporting with detailed information regarding types, positioning, and procedure of implantation. Detailed information regarding the patient's history and supportive information regarding risk factors. A longer follow-up is necessary to evaluate the implant survival further, but at the time of the last follow-up, there were no signs of bleeding or increased probing depths, which indicated good implant health.			

**Study**	Jensen, J. L.; Brox, H. T.; Storhaug, K.; Ambjørnsen, E.; Støvne, S. A.; Bjørnland, T. Dental Implants in Patients with Osteogenesis Imperfecta: A Retrospective and Prospective Study with Review of the Literature. Oral Surgery 2011, 4 (3), 105–114	Yes<br>No<br>Partially	Text reference	Rationale
**Selection**				
Does the patient(s) represent(s) the whole experience of the investigator (center) or is the selection method unclear to the extent that other patients with similar presentation may not have been reported?		Yes	…inclusion criteria were a diagnosis of OI and previous treatment with dental implants (retrospective study), or agenesis or loss of teeth, and that the patients were in need of rehabilitation with dental implants and prosthodontic treatment (prospective study).	Clear inclusion criteria for this case series.
**Ascertainment**				
Was the exposure adequately ascertained?		Yes	Table with detailed implant characteristics for each implant and patient.	Detailed information on position and type of implants and detailed information on surgery procedure.
Was the outcome adequately ascertained?		Yes	At last review, all prosthetic constructions were in place. The total implant survival rate was 95.7%, being 93.3% in the retrospective group and 100%in the prospective group.	Detailed outcome results were described with both bone loss and time of last follow-up.
**Causality**				
Were other alternative causes that may explain the observation ruled out?		Partially	…most implants in the retrospective group were acceptable, only two patients had a mean bone loss of 4 mm or […]. Both patients were smokers.	Not clear if smoking was responsible for the bone loss since both patients report bone loss only on one of their inserted implants.
Was follow-up long enough for outcomes to occur?		Yes	The results of the prospective group, who were followed for a mean period of 1.5 years, were more favorable than those of the retrospective group, followed for a mean time of 6 years.	No sign of significant bone loss in most implants, longer follow up time necessary in prospective cohort to evaluate implant survival further.
**Reporting**				
Is the case(s) described with sufficient details to allow other investigators to replicate the research or to allow practitioners make inferences related to their own practice?		Yes	Multiple (also see the references above).	Detailed information on surgery procedure and materials used was provided.
**Overall rating**	Good quality of reporting with detailed information regarding types, positioning, and procedure of implantation. A longer follow-up is necessary for the prospective cohort to evaluate the implant survival further, but at the time of the last follow-up, there were no increased bone loss in the prospective group and only moderate bone loss in the worst cases of the retrospective group, which indicated good implant health.			

Study	Lee CY, Ertel SK. Bone graft augmentation and dental implant treatment in a patient with osteogenesis imperfecta: review of the literature with a case report. Implant Dent. 2003;12(4):291–5.	Yes<br>No<br>Partially	Text reference	Rationale
**Selection**				
Does the patient(s) represent(s) the whole experience of the investigator (center) or is the selection method unclear to the extent that other patients with similar presentation may not have been reported?		Yes	A 43-year-old Asian woman with a history of OI was referred for evaluation and treatment to replace the missing teeth in the right posterior mandible with implant-supported ceramometal restorations.	Single case report with no indication that other patients with similar presentation may not have been reported.
**Ascertainment**				
Was the exposure adequately ascertained?		Yes	After 4 months of healing, the rigid fixation screws were removed and 2 Paragon […] Screw-vent internal hexed implants were surgically placed in the right mandible under local anesthesia.	Detailed information on position and type of implants and detailed information on surgery procedure.
Was the outcome adequately ascertained?		Partially	The patient continues to return for observation and in the past 2 years, the implants, and prosthesis have remained stable and in function.	Information regarding bone loss or probing depths are missing, only a stable situation is described.
**Causality**				
Were other alternative causes that may explain the observation ruled out?		No	-	No bone loss or loss of implants. Therefore, this domain does not seem to be important for this study.
Was follow-up long enough for outcomes to occur?		Partially	…observation and in the past 2 years…	Stable situation described after 2 years but longer follow up time necessary to evaluate implant survival further.
**Reporting**				
Is the case(s) described with sufficient details to allow other investigators to replicate the research or to allow practitioners make inferences related to their own practice?		Yes	Multiple (also see the references above).	Detailed information on surgery procedure and materials used was provided.
**Overall rating**	Medium quality of reporting. Information regarding clinical bone loss or probing depths are missing. No clear indications what exactly “remained stable” means. Information on type of implants, implantation site and bone augmentation are sufficient for reproducibility.			

Study	Myint M, Støvne SA, Sæves R, Bjørnland T, Jensen JL. Dental implants in individuals with osteogenesis imperfecta: a 6-year follow-up study. Oral Surgery. 2019;12(3):272–7.	Yes<br>No<br>Partially	Text reference	Rationale
**Selection**				
Does the patient(s) represent(s) the whole experience of the investigator (center) or is the selection method unclear to the extent that other patients with similar presentation may not have been reported?		Yes	…aim of the present study was to follow-up our previous study…	Follow-up study with patients from the previous Jensen et al. study (see above).
**Ascertainment**				
Was the exposure adequately ascertained?		Yes	Exposure was ascertained in the previous Jensen et al. study (see above).	Information regarding the implant site, brand and characteristics of the implants are stated.
Was the outcome adequately ascertained?		Yes	Thus, total survival rate of the implants still stands at 100% or at 91% when counting the implant neck fracture.	Detailed information regarding radiological bone loss and time of the last follow-up.
**Causality**				
Were other alternative causes that may explain the observation ruled out?		Yes	One implant was removed after 76 months due to an implant neck fracture, unrelated to disease.	Due to a fracture of one implant the authors declare that the loss of the given implant stands in no connection to OI.
Was follow-up long enough for outcomes to occur?		Yes	…followed up after an average of 93 months…	Nearly 8 years of follow-up with bone loss of 4mm at most indicates good implant survival.
**Reporting**				
Is the case(s) described with sufficient details to allow other investigators to replicate the research or to allow practitioners make inferences related to their own practice?		Yes	Multiple (also see the references above).	Detailed information regarding the follow-up of the previous study by Jensen et al.
**Overall rating**	Good quality of reporting. The given information regarding bone loss of 4mm at most at a follow-up time of nearly 8 years indicates good implant survival. The loss of one implant during follow-up was unrelated to the disease because the loss was associated with an implant neck fracture.			

Study	Payne MA, Postlethwaite KR, Smith DG, Nohl FS. Implant-supported rehabilitation of an edentate patient with osteogenesis imperfecta: a case report. Int J Oral Maxillofac Implants. October 2008;23(5):947–52.	Yes<br>No<br>Partially	Text reference	Rationale
**Selection**				
Does the patient(s) represent(s) the whole experience of the investigator (center) or is the selection method unclear to the extent that other patients with similar presentation may not have been reported?		Yes	A 34-year-old Caucasian woman with osteogenesis imperfecta (type IV) was referred to the implant clinic.	Single case report with no indication that other patients with similar presentation may not have been reported.
**Ascertainment**				
Was the exposure adequately ascertained?		Yes	Branemark System MK III Ti-Unite implants […] were placed (Table with detailed information regarding implant dimensions and positions).	Detailed description of surgical procedure with dimensions and positions of implants.
Was the outcome adequately ascertained?		Yes	No pathologic peri-implant bone loss has been detected…	Detailed information regarding bone loss and maintenance of implants during follow-up appointments are described.
**Causality**				
Were other alternative causes that may explain the observation ruled out?		No	-	No bone loss or loss of implants. Therefore, this domain does not seem to be important for this study.
Was follow-up long enough for outcomes to occur?		Partially	The patient has had clinical and radiographic follow-up for 2 years.	Stable situation described after 2 years, longer follow up time necessary to evaluate implant survival further.
**Reporting**				
Is the case(s) described with sufficient details to allow other investigators to replicate the research or to allow practitioners make inferences related to their own practice?		Yes	Multiple (also see the references above).	Detailed information on surgery procedure and materials used was provided.
**Overall rating**	Good quality of reporting with detailed information regarding surgical procedure, materials used and bone loss at follow-up. A longer follow-up is necessary for final assessment of implant survival.			

**Study**	Prabhu N, Duckmanton N, Stevenson AR, Cameron A. The placement of osseointegrated dental implants in a patient with type IV B osteogenesis imperfecta: a 9-year follow-up. Oral Surg Oral Med Oral Pathol Oral Radiol Endod. March 2007;103(3):349–54.	Yes<br>No<br>Partially	Text reference	Rationale
**Selection**				
Does the patient(s) represent(s) the whole experience of the investigator (center) or is the selection method unclear to the extent that other patients with similar presentation may not have been reported?		Yes	A 32-year-old male patient of oriental origin was referred to our department in 1991 by the clinical geneticist for assessment and management of his dental problems.	Single case report with no indication that other patients with similar presentation may not have been reported.
**Ascertainment**				
Was the exposure adequately ascertained?		Yes	6 maxillary titanium bone tapped Branemark […] implants were placed in the right and left premolar region and a further 5 in the mandibular anterior region.	Detailed description of surgical procedure and type of implants used.
Was the outcome adequately ascertained?		Yes	The final outcome of treatment in the present case has been successful 9 years after initial surgery, with postoperative osseointegration results being similar to what is normally reported in healthy edentulous individuals…	Detailed description of the clinical situation at the time of last follow-up.
**Causality**				
Were other alternative causes that may explain the observation ruled out?		No	The maxillary bone was found to be poor in quality (porous with reduced density) and quantity.	No alternative causes were stated, which could explain the loss of one implant. During implantation the bone was found to be in poor quality.
Was follow-up long enough for outcomes to occur?		Yes	…9 years after initial surgery…	Stable situation after 9 years of follow-up indicates good implant health despite one loss of implant after 12 months.
**Reporting**				
Is the case(s) described with sufficient details to allow other investigators to replicate the research or to allow practitioners make inferences related to their own practice?		Yes	Multiple (also see the references above).	Detailed information on surgery procedure and materials used was provided.
**Overall rating**	Good quality of reporting with a long follow-up period of 9 years. Detailed information regarding the surgical procedure and type of implants was stated. Even though one implant was lost after 12 months, the remaining implants are stable with no loss of bone surround them.			

Study	Prabhu S, Fortier K, May M, Reebye U. Implant therapy for a patient with osteogenesis imperfecta type I: review of literature with a case report. INTERNATIONAL JOURNAL OF IMPLANT DENTISTRY. 23. November 2018;4.	Yes<br>No<br>Partially	Text reference	Rationale
**Selection**				
Does the patient(s) represent(s) the whole experience of the investigator (center) or is the selection method unclear to the extent that other patients with similar presentation may not have been reported?		Yes	A 53-year-old male diagnosed with OI type I was referred to our clinic for extraction of the remaining maxillary teeth and evaluation for full arch immediate load hybrid prosthesis.	Single case report with no indication that other patients with similar presentation may not have been reported.
**Ascertainment**				
Was the exposure adequately ascertained?		Yes	Table with chronological timeline of implant therapy with characteristics of each implant.	Detailed information regarding surgical procedure, type of implant and implant site.
Was the outcome adequately ascertained?		Partially	Probing depths have remained 2–4 mm with no bleeding or purulent drainage at the fixture's sites. […] all healing post-operatively was uneventful.	Information regarding general probing depths is given without specifying probing depths for each individual implant.
**Causality**				
Were other alternative causes that may explain the observation ruled out?		No	-	No loss of implants or significant probing depths. Therefore, this domain does not seem to be important for this study.
Was follow-up long enough for outcomes to occur?		Partially	Our patient is now 4 years post-placement of his first implant procedure and has been functioning without any issues.	Since there are 35 months between the first and the last implant surgery, some of the implants were placed only 13 months prior to the last follow-up.
**Reporting**				
Is the case(s) described with sufficient details to allow other investigators to replicate the research or to allow practitioners make inferences related to their own practice?		Yes	Multiple (also see the references above).	Detailed information on surgery procedure and materials used was provided.
**Overall rating**	Good quality of reporting of the surgical procedures, but some information regarding the follow-up is insufficient. With a range of 13-48 months of follow-up, the implant health of the first implants can be assessed more accurately. Longer follow-up is necessary for final assessment of implant survival.			

**Study**	Wannfors K, Johansson C, Donath K. Augmentation of the mandible via a „tent-pole“ procedure and implant treatment in a patient with type III osteogenesis imperfecta: clinical and histologic considerations. Int J Oral Maxillofac Implants. Dezember 2009;24(6):1144–8.	Yes<br>No<br>Partially	Text reference	Rationale
**Selection**				
Does the patient(s) represent(s) the whole experience of the investigator (center) or is the selection method unclear to the extent that other patients with similar presentation may not have been reported?		Yes	A 30-yearl-old woman diagnosed with type III OI was referred […] to the Department of Oral and Maxillofacial Surgery…	Single case report with no indication that other patients with similar presentation may not have been reported.
**Ascertainment**				
Was the exposure adequately ascertained?		Yes	Four implant sites were prepared with the greatest caution and with awareness of the brittleness of the bone.	Detailed information regarding surgical procedure and type of implant.
Was the outcome adequately ascertained?		Partially	A moderate horizontal reduction in crestal bone was seen…	Reduction of bone was stated, without probing or radiological measurements.
**Causality**				
Were other alternative causes that may explain the observation ruled out?		No	-	No loss of implants or significant bone loss. Therefore, this domain does not seem to be important for this study.
Was follow-up long enough for outcomes to occur?		Partially	…at the 3-year follow-up.	Because of moderate bone loss after 3 years, longer follow-ups may be necessary.
**Reporting**				
Is the case(s) described with sufficient details to allow other investigators to replicate the research or to allow practitioners make inferences related to their own practice?		Yes	Multiple (also see the references above).	Detailed information on surgery procedure and materials used was provided.
**Overall rating**	Good quality of reporting, with detail on histological properties of the patient's bone and surgical procedure. Information regarding clinical bone loss should be more specific and a longer follow-up is necessary for final assessment of implant survival.			

Study	Zola MB. Staged sinus augmentation and implant placement in a patient with osteogenesis imperfecta. J Oral Maxillofac Surg. April 2000;58(4):443–7.	Yes<br>No<br>Partially	Text reference	Rationale
**Selection**				
Does the patient(s) represent(s) the whole experience of the investigator (center) or is the selection method unclear to the extent that other patients with similar presentation may not have been reported?		Yes	…a 25-year-old white man with osteogenesis imperfecta was referred for […] possible placement of osseointegrated implants.	Single case report with no indication that other patients with similar presentation may not have been reported.
**Ascertainment**				
Was the exposure adequately ascertained?		Yes	Multiple paragraphs on different surgical procedures.	Detailed information regarding the placement of 16 implants in total. Information regarding the implant brand is missing.
Was the outcome adequately ascertained?		No	-	Information regarding probing depths or radiological bone loss are missing for all the implants.
**Causality**				
Were other alternative causes that may explain the observation ruled out?		No	…and a radiograph showed fracture of an implant in the maxillary left second premolar area.	The loss of a fractured implant does not seem to stand in a connection with OI.
Was follow-up long enough for outcomes to occur?		Partially	In the ensuing 3 years, the prostheses remained stable…	Some implants were placed after those 3 years of follow-up, without and information regarding further follow-ups.
**Reporting**				
Is the case(s) described with sufficient details to allow other investigators to replicate the research or to allow practitioners make inferences related to their own practice?		Yes	Multiple (also see the references above).	Detailed information regarding the surgical procedure and materials used for bone augmentation.
**Overall rating**	Medium quality of reporting with information regarding the bone loss or probing depths missing. Very detailed description of surgical procedures and materials used for bone augmentation, but only 3 years of follow-up and some implants placed after those 3 years with no follow-up.			
